# Supplementary material for: The C250T Mutation of TERTp Might Grant a Better Prognosis to Glioblastoma by Exerting Less Biological Effect on Telomeres and Chromosomes Than the C228T Mutation
Source: Cancers (Basel). 2024 Feb 9;16(4):735. doi: 10.3390/cancers16040735 (PMC10886885; doi:10.3390/cancers16040735)

C250T mutation of TERTp might grant a better prognosis to glioblastoma by exerting less biological effect on telomeres and chromosomes than the C228T mutation

Teresa Gorria, Carme Crous, Estela Pineda, Ainhoa Hernandez, Marta Domenech, Carolina Sanz, Pedro Jares, Ana Mª Muñoz-Marmol, Oriol Arpí -Llucía, Bárbara Melendez, Marta Gut, Anna Esteve, Anna Esteve-Codina, Genis Parra, Francesc Alameda, Cristina Carrato, Iban Aldecoa, Mar Mallo, Nuria de la Iglesia, Carmen Balana

Supplementary Data

Supplementary Table S1. Progression-free survival (PFS) and overall survival (OS) according to *MGMTp* methylation status and *MGMTp* and *TERTp* status.

Supplementary Figure S1. (A) Progression-free survival and (B) overall survival according to *TERTp* mutation status (wild-type vs mutated)

Supplementary Figure S2. Overall survival according to *TERTp* mutation status (A) C250T mutation vs C228T mutation vs wild-type and (B) C250 mutation vs C228T mutation and wild-type

Supplementary Figure S3. Progression-free survival according to *TERTp* mutation status (A) C250T mutation vs C228T mutation vs wild-type and (B) C250 mutation vs C228T mutation and wild-type

Supplementary Figure S4. TERT RNA expression was assessed in relation to TERTp mutation status using the Kruskal-Wallis test. The results indicated no statistically significant differences across all comparative statuses (C2228T vs C250T vs wt: p=0.25, C228T vs wt: p=0.1, C228T vs C250T: p=0.71, C250T vs wt: p=0.35), as all p-values exceeded the threshold of 0.05.

Supplementary Table S1. Progression-free survival (PFS) and overall survival (OS) according to *MGMTp* methylation status and *MGMTp* and *TERTp* status.

Comparisons		PFS		OS	
		**Months (95% CI)**	***P***	**Months (95% CI)**	***P***
***MGMTp* status**	Met	8.8 (7.1-1.5)	0.002	18.8 (13.9-23.7)	0.023
	UnMet	7.4 (6.5-8.3)		15.8 (13.5-18.1)	
***MGMTp* status & *TERTp* wt vs mut**	Met & wt	7.7 (6.3-16.1)	0.01	13.9 (9.8-NR)	0.146
	Met & C228T+C250T	9.4 (7.8-11.5)		19.6 (14.2-24.6)	
	UnMet & C228T+C250T	7.7 (6.9-9.2)		15.9 (13.7-17.1)	
	UnMet & wt	6.8 (6-1.5)		15 (11.9-27.2)	
***MGMTp* status & *TERTp* C250T vs C228T vs wt**	Met & C250T	12.1 (9.8-22.3)	0.008	24.8 (21.7-NR)	0.021
	UnMet & C250T	7.9 (6.6-13)		18 (13.2-32.7)	
	Met & C228T	8.6 (7.3-1.8)		16 (13.4-21.5)	
	UnMet & C228T	7.7 (6.7-9.4)		14.8 (13.2-16.9)	
	Met & wt	7.7 (6.3-16.1)		13.9 (9.8-NR)	
	UnMet & wt	6.8 (6-1.5)		15 (11.9-27.2)	

Supplementary Figure S1. (A) Progression-free survival and **(B)** overall survival according to *TERTp* mutation status (wild-type vs mutated)

A

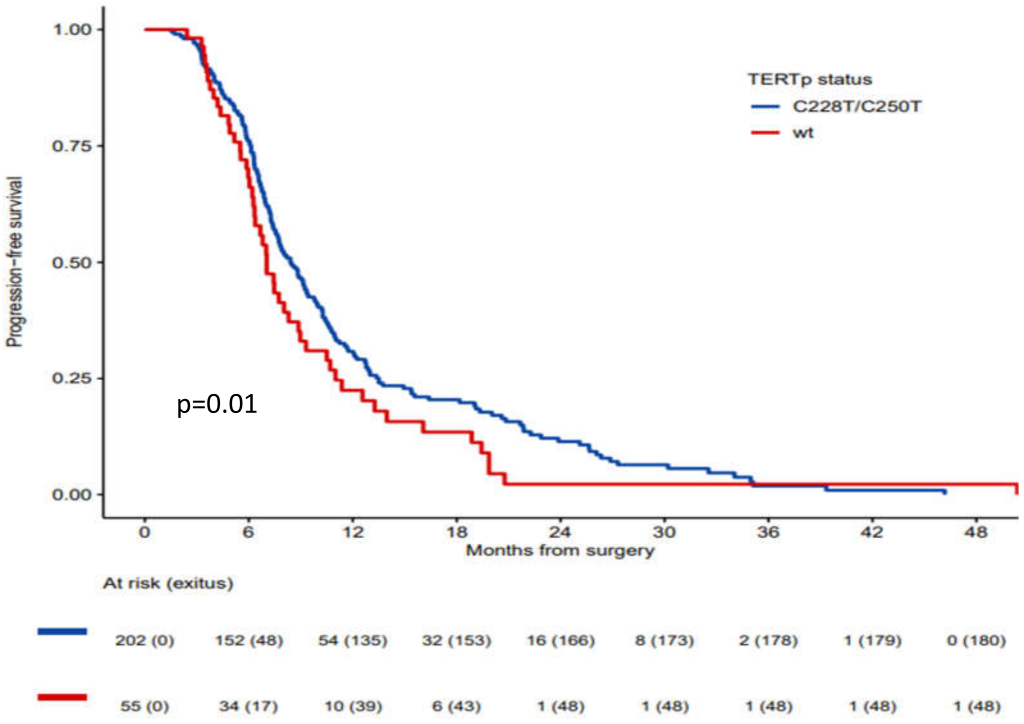

B

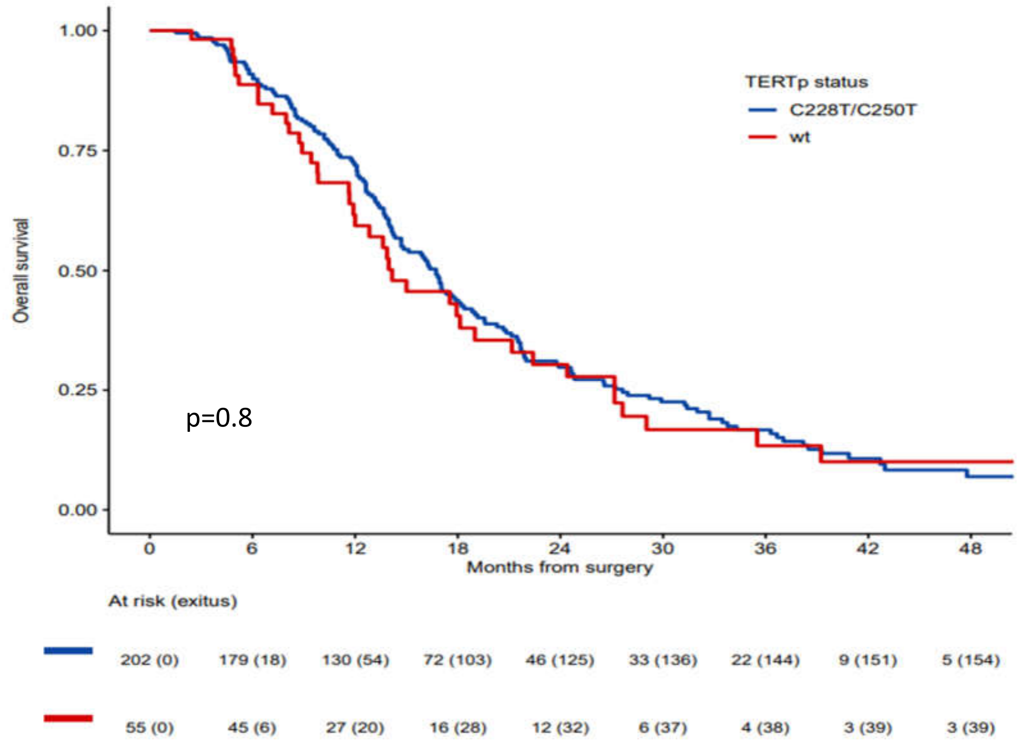

Supplementary Figure S2. Overall survival according to *TERTp* mutation status **(A)** C250T mutation vs C228T mutation vs wild-type and **(B)** C250 mutation vs C228T mutation and wild-type

A

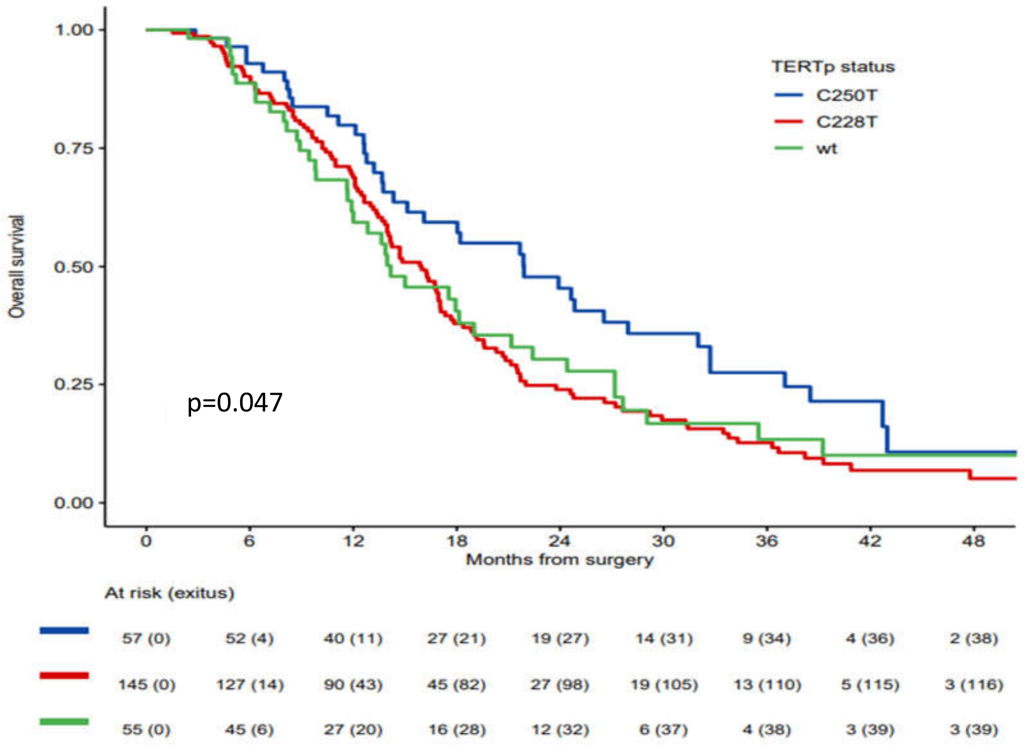

B

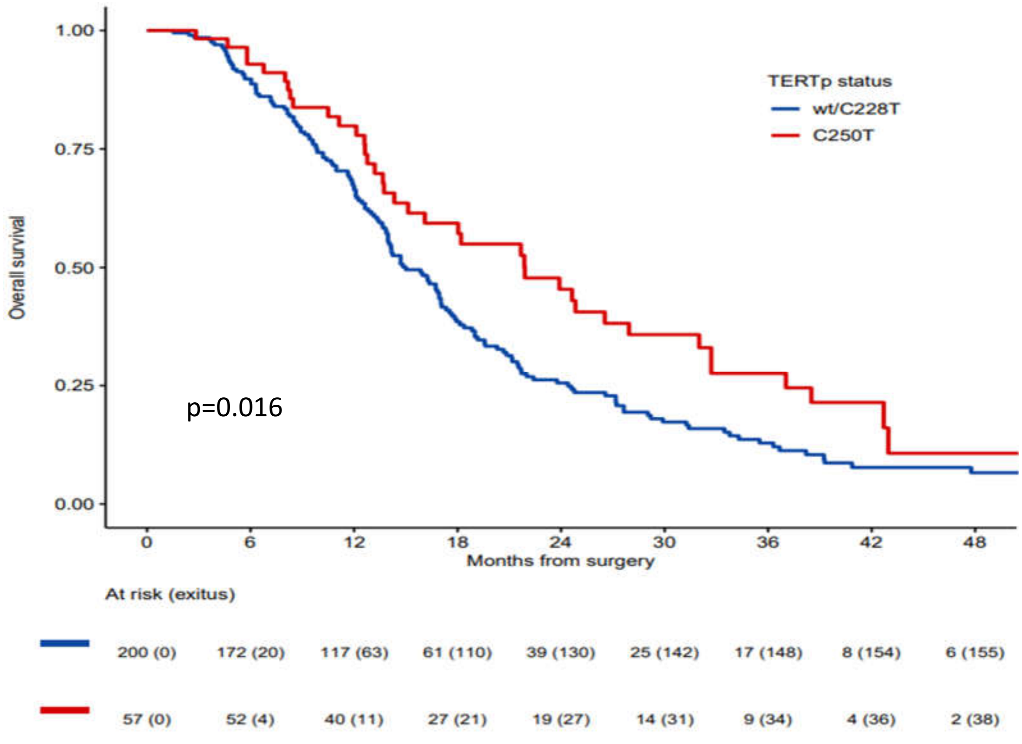

Supplementary Figure S3. Progression-free survival according to *TERTp* mutation status **(A)** C250T mutation vs C228T mutation vs wild-type and **(B)** C250 mutation vs C228T mutation and wild-type

A

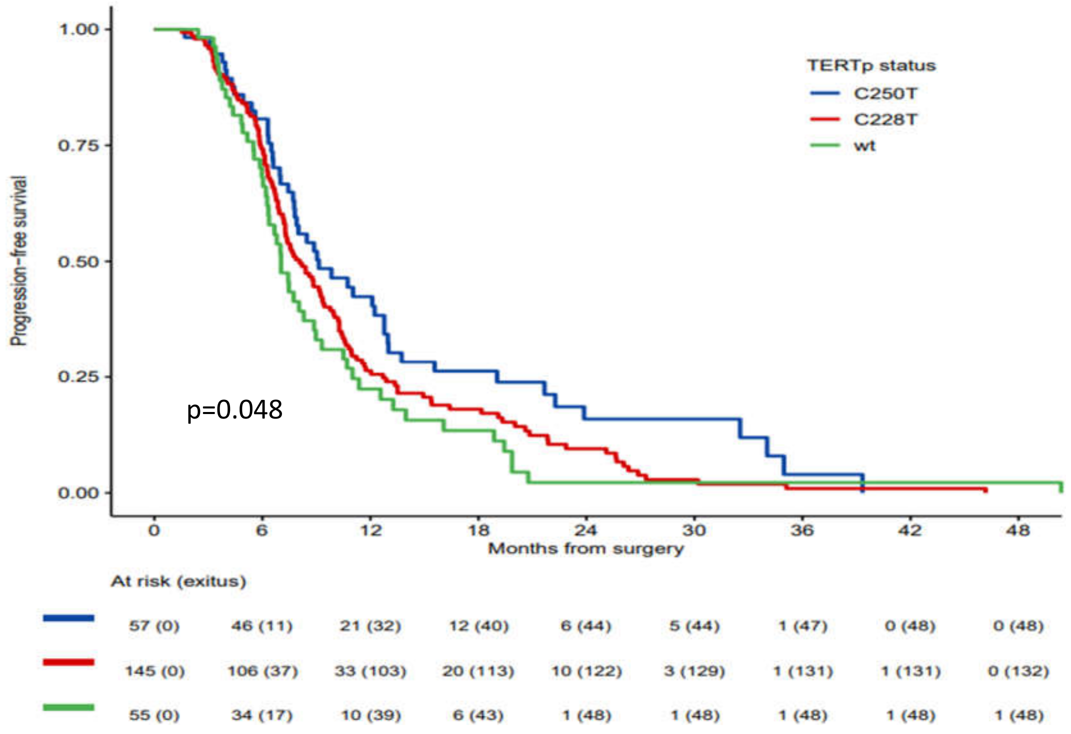

B

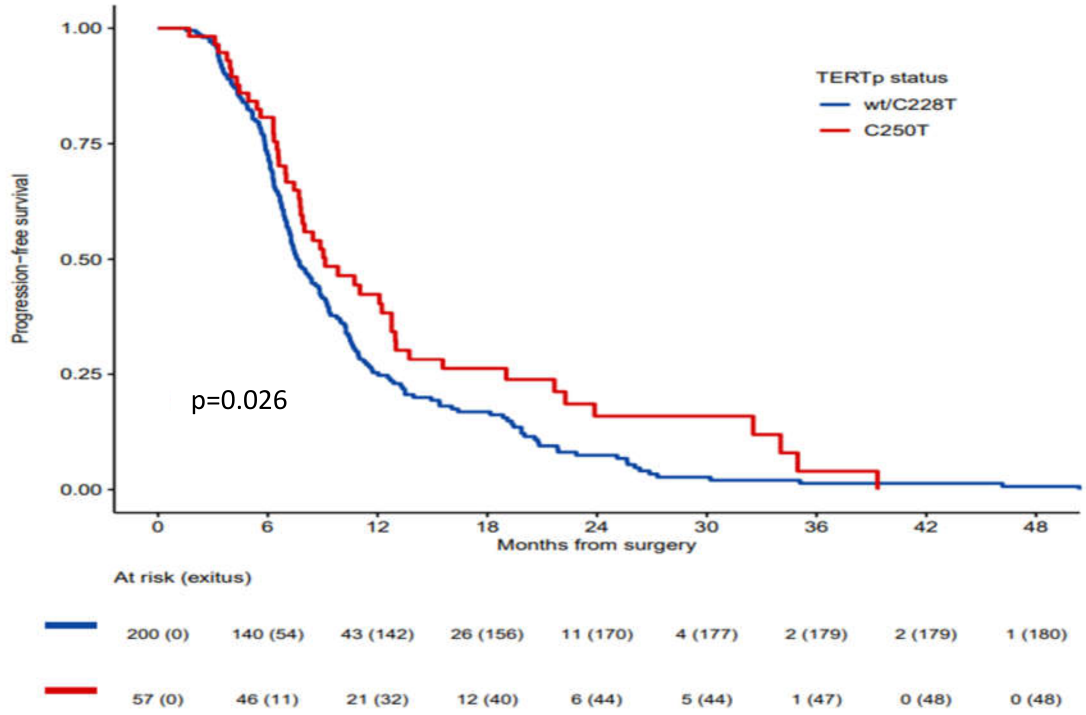

Supplementary Figure S4. TERT RNA expression was assessed in relation to TERTp mutation status using the Kruskal-Wallis test. The results indicated no statistically significant differences across all comparative statuses (C2228T vs C250T vs wt: p=0.25, C228T vs wt: p=0.1, C228T vs C250T: p=0.71, C250T vs wt: p=0.35), as all p-values exceeded the threshold of 0.05.

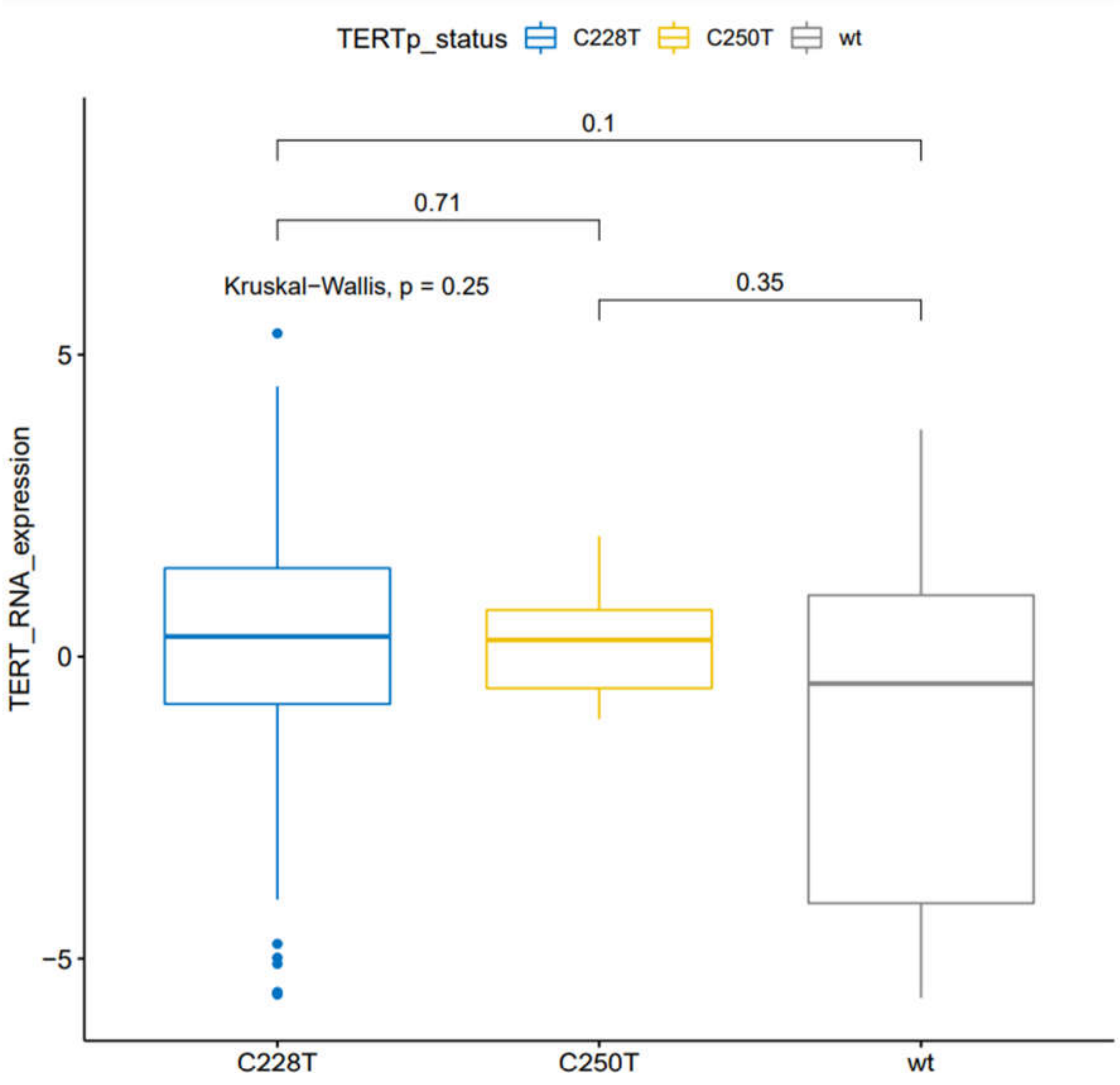

Supplement: Supplementary file 1 [file cancers-16-00735-s001.zip › cancers-2852183-supplementary.pdf]
